# Erythrocyte membrane–liposome coating sustains circulation stability and targeted tumor therapy of CAR-T cells

**DOI:** 10.3389/fimmu.2026.1799107

**Published:** 2026-04-02

**Authors:** Zhiying Chen, Muya Zhou, Xiaoyu Li, Yuqi Zhao, Zhengling Wang, Jinhua Hu, Jialu Hong, Shenrui Zhou, Feiyan Pan, Zhigang Hu, Lingfeng He, Tao Gao, Xiaodong Jiang, Zhiqiang Wang, Zhigang Guo

**Affiliations:** 1 Jiangsu Key Laboratory for Molecular and Medical Biotechnology, College of Life Sciences, Nanjing Normal University, Nanjing, China; 2 Lianyungang First People’s Hospital (Affiliated Lianyungang Hospital of Xuzhou Medical University, Lianyungang Clinical College of Nanjing Medical University), Lianyungang, China; 3 The Second Hospital of Tianjin Medical University, Tianjin, China

**Keywords:** cancer immunotherapy, CAR-T cells, non-target sequestration, red blood cell membrane liposomes, tumor targeting

## Abstract

**Background:**

CAR-T therapy is limited by off-target sequestration in liver, spleen, and lungs, which reduces tumor delivery and risks systemic toxicity. Genetic engineering approaches are complex and carry safety concerns, necessitating efficient non-genetic strategies to optimize CAR-T biodistribution.

**Purpose:**

We developed red blood cell membrane–chimeric liposomes (Rlip) as a biomimetic coating system to non-genetically modify CAR-T cells, aiming to enhance immune evasion, prolong circulatory persistence, and redirect trafficking toward tumors while preserving intrinsic effector function.

**Methods:**

Rlip-CAR-T cells were prepared via simple incubation. *In vitro* characterization assessed coating stability, CD47-mediated macrophage resistance, phenotype preservation, and antigen-specific cytotoxicity. *In vivo* studies evaluated biodistribution, tumor infiltration, and efficacy in Nalm-6 systemic leukemia and 1806-luc subcutaneous ovarian cancer models.

**Results:**

Rlip coating stably presented CD47, functionally impairing macrophage phagocytosis and enhancing peripheral persistence without altering memory subsets, activation markers, or cytotoxicity. Rlip-CAR-T cells demonstrated markedly reduced off-target organ accumulation and increased intratumoral enrichment in both models, with superior tumor control and prolonged survival observed in the leukemia model.

**Conclusions:**

Rlip surface engineering represents a universal, clinically translatable strategy to overcome CAR-T biodistribution barriers. By integrating erythrocyte-mimetic immune evasion with optimized tumor delivery, this approach improves CAR-T cell accumulation at tumor sites across hematologic and solid malignancies.

## 1 Introduction

Chimeric antigen receptor T-cell (CAR-T) therapy has achieved remarkable efficacy in the treatment of hematologic malignancies, including relapsed/refractory B-cell acute lymphoblastic leukemia (1) and diffuse large B-cell lymphoma (2); it has also demonstrated therapeutic potential in solid tumors such as lung, breast, liver, and pancreatic cancers (3–6), and has thus become a major focus of ongoing research. In both hematologic and solid malignancies, however, non-target organ sequestration following CAR-T cell infusion constitutes a fundamental hurdle that limits therapeutic efficacy.Therefore, patients require intravenous infusion of large numbers of highly activated engineered cells, which increases the cost of cell manufacturing as well as the risk of developing T-cell toxicity and cytokine release syndrome (CRS).Thereby markedly restricting the clinical applicability of CAR-T therapy utilizing intravenous infusion (7, 8).

Non-target organ retention constitutes an intrinsic targeting deficiency in CAR-T cells after intravenous infusion, attributable to aberrant immune recognition and dysregulated migration. Its mechanisms and detrimental therapeutic consequences have been elucidated in pre-clinical and clinical studies (9–12). At the molecular level, the principal trigger is non-specific recognition by the mononuclear-phagocyte system (MPS). Although CAR-T cells are derived from autologous T lymphocytes, *in-vitro* activation and expansion down-regulate the “don’t-eat-me” signal CD47, rendering them susceptible to scavenger receptors expressed on hepatic sinusoidal endothelial cells and splenic macrophages (10, 13, 14). Studies have shown that, following infusion, the majority of CAR-T cells are sequestered in the spleen and lymph nodes, whereas a smaller fraction accumulates in the lungs and liver (15–18). This sequestration markedly reduces the fraction of functional CAR-T cells that reach target tumors. Consequently, clinicians are compelled to administer higher CAR-T cell doses to achieve therapeutically effective intra-tumoral concentrations. Multiple clinical investigations have unequivocally demonstrated a positive correlation between CAR-T-cell dose and both the incidence and severity of CRS. High-dose infusions amplify the cascading release of pro-inflammatory cytokines such as IFN-γ and IL-6 after CAR-T activation, thereby exacerbating systemic inflammation (19, 20).

Given that CAR-T cells must precisely home to tumor sites after intravenous infusion, their targeted distribution within the body directly impacts therapeutic efficacy and safety. Reducing non-targeted retention is essential for improving CAR-T therapy outcomes. Several research groups have thus been addressing this problem by focusing on cell migration regulation and structural optimization. For example, Wang H et al. achieved a significant reduction in off-target toxicity while preserving tumor-killing activity in mouse models through the knockout of CD11a, CD49d, and PSGL1 in T cells, thereby inhibiting their adhesion to and migration into normal tissues (21). Additionally, a structural modification strategy has been applied. For instance, Muthuvel M et al. incorporated the CD8α hinge region, which reduces non-specific binding of CAR-T cells to MHC class I molecules, thereby mitigating the risk of non-specific inflammation triggered by off-target activation (22). Other studies have engineered synthetic intramembrane proteolysis receptors that enforce CAR expression exclusively within tumors through dual-antigen logic gating, thereby eliminating off-target sequestration of CAR-T cells in normal organs (23). While these strategies offer effective insights into reducing off-target toxicity and preserving antitumor activity in CAR-T cells, current approaches predominantly rely on genetic editing or complex CAR structural modifications. These methods entail technically cumbersome workflows, high clinical translation costs, concerned biosafety and lack a universal *in vivo* distribution modulation strategy that addresses the distinct microenvironmental features. Therefore, developing an efficient distribution strategy of CAR-T cells that integrates safety, universality, and technical feasibility is crucial for advancing clinical translation, and this objective represents a critical focus of the present study.

To address these limitations, we pursued a non-genetic, biomimetic strategy to broadly optimize CAR-T cell distribution while preserving their intrinsic function. Liposomal nanocarriers, with established biocompatibility and clinical translatability, provide an ideal scaffold for integrating biological membrane components (24–26). By incorporating red blood cell (RBC) membrane-derived coatings, these hybrid vesicles display universal “don’t-eat-me” signals such as CD47, which inhibit phagocytic clearance by the mononuclear phagocyte system (27–29). This mechanism is expected to prolong CAR-T cell circulation time and minimize non-specific sequestration in off-target organs. This coating strategy circumvents the technical complexity and high costs of genetic manipulation, offering a broadly applicable solution for both hematologic and solid tumor settings. We therefore hypothesized that RBC membrane–liposome surface engineering could serve as a safe, universal, and technically feasible approach to enhance CAR-T cell pharmacokinetics and biodistribution, thereby facilitating improved intratumoral infiltration and antitumor efficacy.

Here, we have constructed an RBC membrane–chimeric liposome (Rlip) for surface engineering of CAR-T cells (Rlip-CAR-T), and thus systematically evaluated the efficacy and feasibility of Rlip-CAR-T distribution through *in vitro* and *in vivo* experiments. *In vitro* analyses revealed that the chimeric liposomes coating does not compromise the core functional phenotypes and cytotoxicity of CAR-T cells. *In vivo* studies demonstrated beneficial effects in both hematologic and solid tumor models. Rlip-CAR-T cells exhibited significantly higher circulating numbers in blood, reduced off-target sequestration, and enhanced antitumor efficacy in both models, and significantly increased intratumoral CAR-T accumulation in the solid tumor model. This investigation follows the rationale of preserving functional integrity, optimizing biodistribution, and enhancing tumor targeting, thereby simultaneously addressing therapeutic demands in both hematologic and solid malignancies and showing considerable promise for extending CAR-T therapy to solid tumors.

## 2 Materials and methods

### 2.1 Chemicals

1,2-Dioctadecanoyl-sn-glycero-3-phospho-(1’-rac-glycerol) (sodium salt) (DSPG), 1,2-distearoyl-sn-glycero-3-phosphoethanolamine (DSPE) were purchased from Aladdin Holdings Group Co., Ltd. (Beijing, China), Cholesterol was purchased from MedChemExpress. 1,2-Distearoyl-sn-glycero-3-phosphoethanolamine-polyethylene glycol-phenylboronic acid)(DSPE-PEG-PBA) was purchased from Guangzhou Weihua Biotechnology Co., Ltd. (Guangzhou, China). Rhodamine B (RhB) was purchased from Shanghai Aladdin Biochemical Technology Co., Ltd. The antibodies used in this study, including APC-anti-CD19, APC-anti-PD-1, FITC-anti-LAG-3, PE-anti-CD62L, APC-anti-CD45RA, PE-anti-CD69, and APC-anti-CD25, were all obtained from Biolegend. For the detection of human anti-MSLN CAR and anti-CD19 CAR, the reagents used were PE-labeled human MSLN protein (KACTUS) and APC-labeled human CD19 protein (Hys-bio), respectively.

### 2.2 RBC membrane isolation

Human blood samples used in this study were provided by Taikang Xianlin Drum Tower Hospital. After transferring the collected blood into centrifuge tubes, it was centrifuged at 3000 rpm for 20 min in a refrigerated centrifuge at 4 °C to pellet red blood cells (RBCs). Subsequently, the plasma and the flocculent precipitate on the surface of the RBCs pellet were aspirated and discarded to avoid contamination with other cell types. Five volumes of normal saline were added to the RBC layer, followed by gentle inversion to mix thoroughly. The mixture was then centrifuged at 1500×g for 10 min at 4 °C, and this washing step was repeated twice to obtain purified RBCs. A 50 μL aliquot of purified RBCs was mixed with 1 mL ice-cold PBS (0.25X) to lyse the RBCs via hypotonic treatment. After lysis, the sample was centrifuged at 10000×g for 15 min at 4 °C, and the supernatant containing hemoglobin and other cellular components was discarded. Next, 1 mL ice-cold PBS (0.25X) was added to the pellet for gentle resuspension, and the sample was centrifuged again at 10000×g for 15 min at 4 °C. This washing step was repeated several times, ultimately yielding a white pellet containing RBC ghosts. Finally, the concentration of the pellet was adjusted to 100 μg/mL using PBS (1X).

### 2.3 Fabrication of RBC membrane-liposome and physicochemical characterization

RBC membrane-liposome vesicles were prepared by a classical thin-film hydration protocol followed by extrusion-assisted membrane fusion. To fine-tune surface charge and colloidal stability, five lipid formulations (Rlip-1–5) were designed. For each formulation, the lipid mixture and 1 mol % RhB were co-dissolved in chloroform. The solvent was removed under a gentle nitrogen stream to yield a uniform thin film. The film was hydrated with 800 μL of phosphate-buffered saline (PBS), after which 80 μg of RBC membrane (30) was added. The suspension was vortex-mixed thoroughly and sonicated in a water bath for 5 min to achieve homogeneity. Six freeze–thaw cycles (liquid nitrogen ↔ 37 °C water bath) were then applied. Subsequently, the dispersion was sequentially extruded 21 times through a 1 μm polycarbonate membrane, followed by filtration through 0.8 μm and 0.2 μm membranes, yielding RhB-labeled, RBC-membrane-camouflaged liposomes with a narrow size distribution.

Subsequently, the hydrodynamic size and surface charge of the BML were characterized using a dynamic light scattering (DLS) analyzer (Malvern Zetasizer Nano ZSP), and the zeta potential was monitored to ensure the consistency of the preparation process.

### 2.4 Transmission electron microscope

For negative-stain TEM imaging, the method was performed as previously reported (31). Briefly, a 5 μL aliquot of the sample was applied onto a carbon-coated 200-mesh copper grid and allowed to adsorb for 3–5 min. Excess liquid was carefully blotted with filter paper. Subsequently, the grid was stained with 2% (w/v) phosphotungstic acid for 2–3 min, followed by removal of the surplus stain using filter paper. After air-drying at room temperature, the specimens were examined under a transmission electron microscope (TEM), and micrographs were acquired for image analysis.

### 2.5 Cell lines and culture

Human breast cancer cell line HCC1806, ovarian cancer cell line OVCAR3, B-cell lymphoma cell line Raji, human monocytic leukemia cell line THP-1, and B-cell precursor leukemia cell line NALM-6 were purchased from the American Type Culture Collection (ATCC). HCC1806, OVCAR3, Raji, and NALM-6 stably expressing firefly luciferase (ffluc) were obtained by lentiviral transduction and designated HCC1806-luc, OVCAR3-luc, Raji-luc, and NALM-6-luc, respectively.THP-1-GFP cells stably expressing green fluorescent protein (GFP) were established by lentiviral transduction. HEK293T cells were cultured in DMEM (Gibco) supplemented with 10% FBS. OVCAR3-luc, HCC1806-luc, Raji-luc, and NALM-6-luc cells were cultured in RPMI-1640 (Gibco) supplemented with 10% FBS. All cells were maintained at 37 °C in a humidified incubator with 5% CO_2_.

### 2.6 The generation of CAR-T cells

CAR-T cells were generated as previously described (32). Briefly, chimeric antigen receptors targeting MSLN or CD19 were constructed by fusing our in-house-selected fully human scFvs or the canonical FMC63 scFv with a CD8α leader peptide, hinge and transmembrane domains, and a 4-1BB/CD3ζ signaling tail; the resulting cassette was cloned into the pCDH lentiviral backbone. Primary T lymphocytes were isolated from peripheral blood mononuclear cells (PBMCs; HYCELLS, hPB050C) by negative selection using the EasySep™ Human T-Cell Isolation Kit (Stemcell, 17951), activated with Dynabeads™ Human T-Expander CD3/CD28 beads (Thermo Fisher Scientific, 11141D) at a 1:1 bead-to-cell ratio, and transduced 24 h later with VSV-G-pseudotyped lentiviral particles encoding the CAR (produced in HEK293T cells) to generate CAR-T cells directed against MSLN or CD19, respectively. Transduced cells were expanded in X-VIVO medium (LONZA) supplemented with 100 IU/mL IL-2; half-medium exchanges were performed every 2–3 days to maintain a density of 0.5–1 × 10^6^ cells/mL.

### 2.7 Assessment of Rlip stability on CAR-T cell surface

To evaluate the stability of Rlip on the CAR-T cell surface, CAR-T cells were first co-incubated with RhB-labeled Rlip at 37 °C for 45 minutes, followed by washing with PBS to remove unbound Rlip, thus obtaining Rlip-CAR-T cells. The Rlip-CAR-T cells were then resuspended in complete medium and incubated at 37 °C with 5% CO_2_ for various durations (0, 24, and 48 hours). At each designated time point, cells were collected, washed with ice-cold PBS, and subjected to nuclear staining with DAPI. Subsequently, cells were fixed with 4% paraformaldehyde (PFA) at room temperature for 10 minutes. The fixed cells were immediately subjected to confocal laser scanning microscopy imaging.

### 2.8 Flow cytometry

1 × 10^5^ cells were collected and washed with PBS to remove the culture medium, then resuspended in 100 μL of PBS. The cells were stained with the following antibodies: APC-anti-CD19, APC-anti-PD-1, FITC-anti-LAG-3, PE-anti-CD62L, APC-anti-CD45RA, PE-anti-CD69, and APC-anti-CD25 (all from Biolegend). For detection of CAR-positive cells, PE-labeled human MSLN protein and APC-labeled human CD19 protein were used to stain anti-MSLN CAR and anti-CD19 CAR T cells, respectively. After adding the antibodies or detection reagents, the cells were vortexed briefly and incubated in the dark at room temperature for 20 minutes. Following incubation, cells were washed twice with 1 mL of PBS (centrifugation at 500 × g for 5 min) to remove unbound antibodies. Finally, the cells were resuspended in 300 μL of PBS and acquired on a CytoFLEX S flow cytometer (Beckman Coulter). Data were analyzed using CytExpert software.

### 2.9 Macrophage phagocytosis detection

Human THP-1-GFP monocytes were differentiated into macrophages by treatment with 100 ng/mL phorbol 12-myristate 13-acetate (PMA) for 48 hours. CAR-T or Rlip-CAR-T cells were labeled with CellTrace Far Red according to the manufacturer’s instructions. Differentiated macrophages (GFP-positive) were then co-cultured with CellTrace Far Red-labeled CAR-T or Rlip-CAR-T cells at a 1:1 ratio in 200 μL of complete medium for 6 hours at 37 °C. After incubation, cells were washed twice with PBS to remove unbound cells, resuspended in fresh PBS, and analyzed by flow cytometry. Macrophages that had phagocytosed the target CAR-T cells were identified as the GFP and CellTrace Far Red double-positive cell population.

### 2.10 Cytotoxicity assays

In the cytotoxicity assay, liposome-modified CAR-T cells were co-cultured with tumor cells at different effector-target (E: T) ratios for 24 hours, and cytotoxicity was subsequently detected. Cytotoxicity of the CAR-T cells was assessed using the One-Lite Luciferase Assay System (Vazyme, DD1203-02) in strict accordance with the manufacturer’s instructions. The cell killing rate was calculated using the following formula: Killing rate = [(Control value - Experimental value)/Control value] × 100%.

### 2.11 Animal experiments

Animal experiments were conducted in the Animal Laboratory of Nanjing Normal University, strictly following the protocol approved by the Animal Welfare Committee of the university (Approval No.: 2020-0047). Eight-week-old NXG mice were purchased from Hangzhou Qizhen Laboratory Animal Science and Technology Co., Ltd. and used in the experiment. Two tumor models were established via different inoculation routes: each mouse received a subcutaneous injection of 2×10^6^ HCC1806-luc cells or an intravenous injection of 1×10^6^ Nalm6-luc cells. CAR-T cell therapy was initiated at different time points based on the tumor model type: therapy for the 1806 cell-derived subcutaneous tumor model was started 14 days post-inoculation, whereas therapy for the Nalm6 cell-derived intravenous tumor model was initiated 9 days post-inoculation. For CAR-T treatment, each mouse was injected with 100 μl of CAR-T cells via the tail vein, with two dose groups set: 1×10^6^ CAR-T cells per mouse (for evaluating tumor regression) and 2×10^6^ CAR-T cells per mouse (for observing off-target organ sequestration). During the experiment, tumor volume and body weight of mice inoculated with 1806 cells were regularly measured and recorded every other day. Tumor volume was calculated using the formula: (length × width²)/2. For mice inoculated with Nalm6 cells, bioluminescence imaging (BLI) was performed on days 6, 15, and 26 post-CAR-T cell therapy. Additionally, for mice in the off-target organ sequestration observation group, blood samples were serially collected on days 1–3 after CAR-T cell infusion, and tissue samples were collected on day 3 post-infusion. All collected samples were subsequently subjected to flow cytometry and immunohistochemical (IHC) analysis to determine relevant indicators.

### 2.12 Analysis of tissue samples

Immunohistochemistry (IHC) was performed by AiFang Biological. For histopathological assessment, murine tissues were fixed in 4% paraformaldehyde, paraffin-embedded, sectioned at 4 μm, and stained with hematoxylin and eosin (H&E). For immunostaining, sections were deparaffinized, rehydrated, subjected to antigen retrieval, and blocked with 3% BSA. Slides were then incubated overnight at 4 °C with an anti-human CD45 primary antibody, followed by secondary antibody incubation, hematoxylin counterstaining, and mounting. Whole-slide images were acquired using a digital slide scanner and analyzed with ImageJ.

### 2.13 Statistical analysis

All data analyses and graphical presentations were executed using GraphPad Prism version 10.1.2. Statistical significance was assessed by means of two-tailed unpaired Student’s t-tests, one-way analysis of variance (ANOVA), or two-way ANOVA, as dictated by experimental design. A threshold of P < 0.05 was considered statistically significant. Data are reported as mean ± standard deviation (SD) unless otherwise specified.

## 3 Results

### 3.1 Synthesis and characterization of the nanoparticles

**Figure 1:**
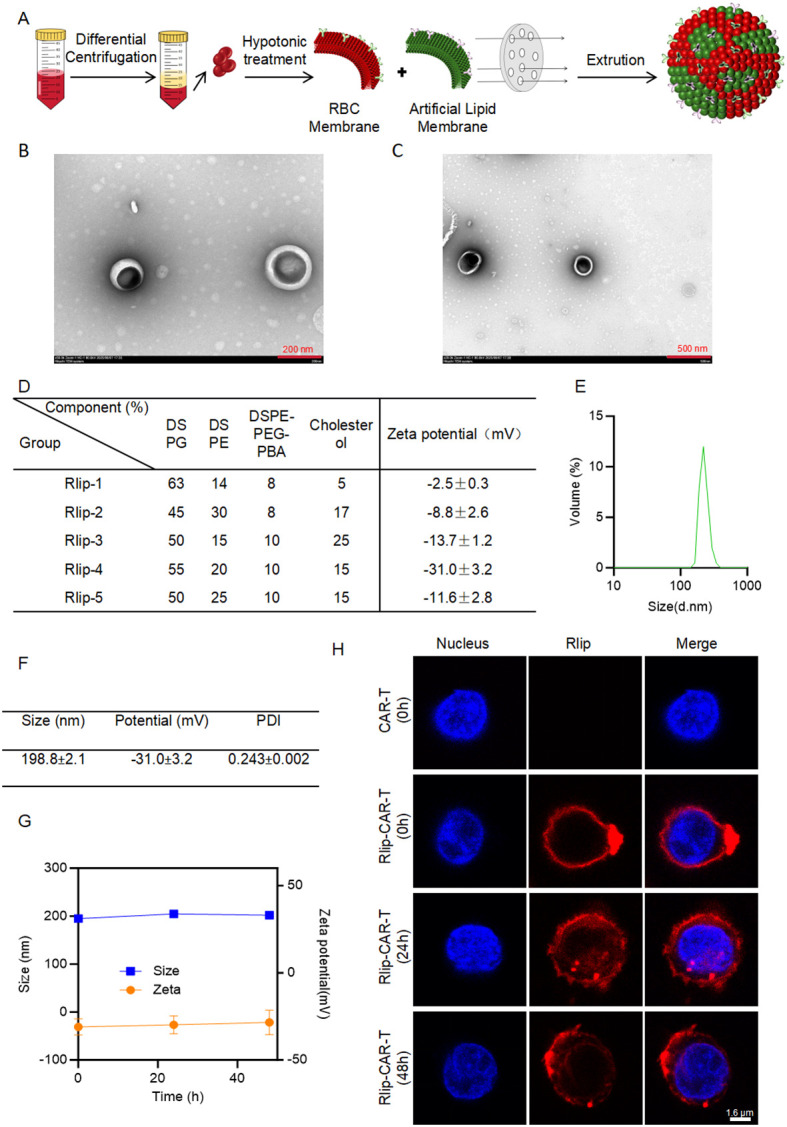
Preparation and characterization of red blood cell membrane–chimeric liposomes (Rlip) nanoparticles. **(A)** Schematic of Rlip nanoparticle preparation. **(B)** and **(C)** TEM images of Rlip-4 at two magnifications (scale bars: 200 nm and 500 nm). **(D)** Zeta potential of four Rlip formulations (mean ± SD, n = 3). **(E)** Size distribution of Rlip-4 (mean ± SD, n = 3). **(F)** Key parameters of Rlip-4 (mean ± SD, n = 3). **(G)** Stability of Rlip-4 over 48 h (mean ± SD, n = 3). **(H)** Confocal microscopy images showing the stability of Rlip (red) on the CAR-T cell surface at different time points. Scale bar: 1.6 μm.

To lay a solid foundation for downstream *in vitro* and *in vivo* studies, we first constructed and characterized Rlip. Since particle size, morphology, and colloidal stability govern surface-engineering efficiency and the *in vivo* fate of CAR-T cells, Rlip was fabricated by a classical thin-film hydration–extrusion protocol (**Figure 1A**). Briefly, erythrocyte membranes were first isolated; a lipid mixture was then dissolved in chloroform and dried under a gentle nitrogen stream to form a thin lipid film. After the addition of PBS containing the isolated erythrocyte membranes, the film was hydrated and the resulting dispersion was extruded through polycarbonate membranes to yield the final nanovesicles. Transmission electron microscopy (TEM) images (**Figures 1B, C**) visually confirm the successful formation of Rlip nanoparticles, revealing their characteristic spherical vesicular morphology and providing a structural basis for subsequent physicochemical characterization.

To identify the optimal formulation, Rlip variants with different molar compositions were prepared, and their zeta potentials were measured (**Figure 1D**). Among them, Rlip-4 exhibited the most negative zeta potential (−31.0 ± 3.2 mV), indicative of superior colloidal stability. DLS analysis showed a monodisperse size distribution for Rlip-4, with a single narrow peak centered at ~200 nm (**Figure 1E**). Quantitative assessment of key physicochemical parameters (**Figure 1F**) gave an average hydrodynamic diameter of 198.8 ± 2.1 nm, a zeta potential of −31.0 ± 3.2 mV, and a polydispersity index (PDI) of 0.243 ± 0.007, corroborating the uniform size distribution and stable surface negative charge of Rlip-4.

The colloidal stability of Rlip-4 was further evaluated by monitoring its size and zeta potential over 48 h (**Figure 1G**). Neither parameter showed any significant fluctuation during the observation period, demonstrating the excellent long-term stability of Rlip-4 under the experimental conditions.Building upon these findings, we further validated the binding stability of Rlip on the CAR-T cell surface. Confocal microscopy observation demonstrated that the liposomal fluorescence remained stably associated with the CAR-T cell surface for at least 48 hours under *in vitro* culture conditions (**Figure 1H**). Collectively, both Rlip and Rlip-CAR-T exhibited desirable stability, establishing a solid foundation for subsequent functional experiments.

### 3.2 Rlip nanoparticles do not alter CAR expression, activation, exhaustion, or cytotoxic function in CD19 CAR-T cells

**Figure 2:**
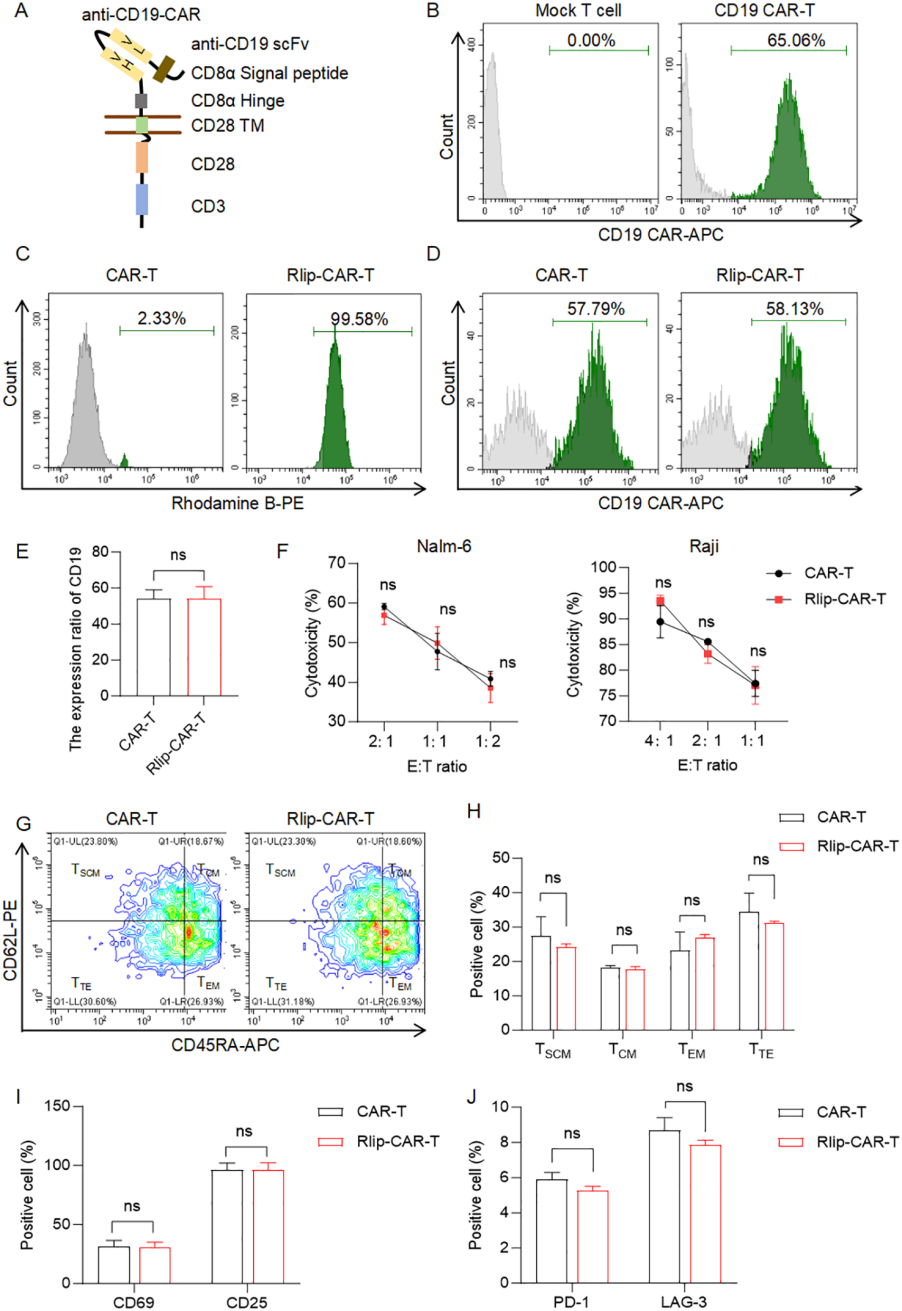
Impact of Rlip modification on CD19 CAR-T cell binding efficiency, CAR expression, and functional phenotype. **(A)**. Schematic of the anti-CD19 CAR construct. **(B)**. Flow-cytometry histograms showing CD19 CAR expression in untransduced human T cells versus anti-CD19 CAR-T cells. (C). Flowcytometry histograms of RhB positivity in CAR-T and Rlip-CAR-T cells (reflecting Rlip binding efficiency). **(D)**. Flow-cytometry dot plots showing CD19 CAR expression levels in CAR-T and Rlip-CAR-T cells. **(E)**. Quantitative analysis of CD19 CAR expression ratios between CAR-T and Rlip-CART cells. **(F)**. Cytotoxic activity of CAR-T and Rlip-CAR-T cells against Nalm-6 and Raji cells at the indicated E:T ratios. **(G)**. Flow-cytometry dot plots showing memory-phenotype distribution of CAR-T and Rlip-CAR-T cells stained for CD62L and CD45RA. **(H)**. Quantitative analysis of T-cell memory subsets (TSCM, TCM, TEM, TTE) in CAR-T and Rlip-CAR-T cells. **(I)**. Quantitative analysis of activation-marker (CD69, CD25) expression in CAR-T and Rlip-CAR-T cells. **(J)**. Quantitative analysis of exhaustion-marker (PD-1, LAG-3) expression in CAR-T and Rlip-CAR-T cells. All data are obtained from at least three donors and presented as mean ± SD; ns, not significant. Cytotoxic activity **(F)** was analyzed by two-way ANOVA; all other quantitative comparisons **(E, H–J)** used two-tailed unpaired t-tests.

To investigate the impact of Rlip nanoparticles on CD19 CAR-T cells, we first generated CD19 CAR-T cells using a validated anti-CD19 CAR construct encoding the FMC63 scFv (**Figure 2A**). Flow cytometry revealed that 65.06% of the cells were CD19 CAR-positive (**Figure 2B**). After incubation with RhB-labeled Rlip for 45 min, 99.58% of the cells became RhB-positive (**Figure 2C**), demonstrating efficient Rlip binding.

To determine whether Rlip affects CAR expression, we compared CD19 CAR positivity between Rlip-treated and untreated CAR-T cells. Flow cytometry showed no significant difference (**Figures 2D, E**), indicating that Rlip binding does not alter CD19 CAR expression.

We next evaluated CAR-T cell function. Cytotoxicity assays using Nalm-6 and Raji cells as targets at various effector-target (E:T) ratios revealed comparable killing activity between Rlip-treated and control CAR-T cells (**Figure 2F**). Memory-subset analysis based on CD45RA/CD62L expression showed no significant differences in T stem-cell memory (TSCM), central memory (TCM), or other subsets (**Figures 2G, H**). Similarly, expression of activation markers CD69 and CD25 (**Figure 2I**) and exhaustion markers PD-1 and LAG-3 (**Figure 2J**) did not differ significantly between the two groups.

Collectively, Rlip nanoparticles bind efficiently to CD19 CAR-T cells without affecting CD19 CAR expression, cytotoxic activity, memory phenotype, activation status, or exhaustion profile, indicating that Rlip does not interfere with the core functions of CAR-T cells.

### 3.3 Rlip anchoring prolongs circulation and enhances *in vivo* antitumor activity of CD19 CAR-T cells

**Figure 3:**
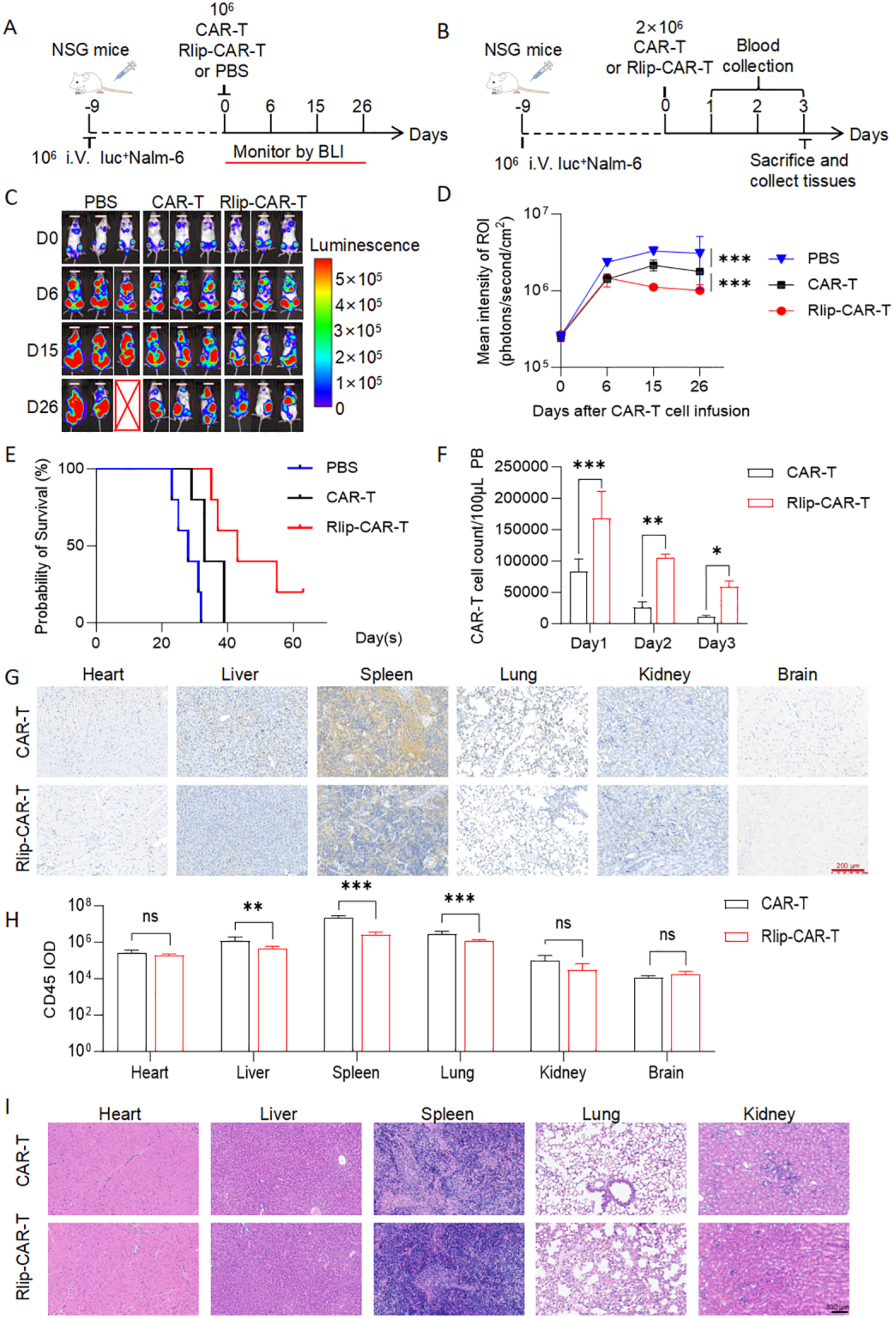
*In vivo* efficacy, peripheral blood kinetics, and tissue distribution of Rlip-CAR-T cells in a Nalm6 xenograft model. **(A)**. Timeline of the *in vivo* experiment: NXG mice bearing systemic Nalm6 leukemia received CAR-T cells, Rlip-CAR-T cells, or PBS, and tumor burden was monitored by bioluminescence imaging (BLI) at indicated time points. N = 5 mice for the PBS, CAR-T and Rlip-CAR-T groups. **(B)**. Sample-collection protocol: serial blood draws after infusion, with tissue harvest on day 3. **(C)**. Representative BLI imaged mice from each group at different time points; the pseudo-color scale indicates tumor load. **(D)**. Quantification of average radiance in BLI regions of interest (ROI). **(E)**. Survival probability curves for each group. **(F)**. Flow-cytometric enumeration of CAR-T cells in peripheral blood on days 1–3 post-infusion (n = 5). **(G)**. Representative CD45 IHC images of major organs; scale bar: 200 μm. **(H)**. Integrated optical density (IOD) quantification of CD45 IHC in major organs. **(I)**. Representative H&E images of major organs showing no obvious pathology; scale bar: 100 μm. All data are presented as mean ± SD; ns, not significant; *p < 0.05; **p < 0.01; ***p < 0.001. **(D)** was analyzed by one-way ANOVA, **(F)** by repeated-measures two-way ANOVA with Bonferroni post-tests, and **(H)** by two-tailed unpaired t-test.

To evaluate the *in vivo* efficacy, peripheral blood abundance, and off-target accumulation of Rlip-CAR-T cells in hematologic malignancies, we established a systemic Nalm6 leukemia model in 8-week-old NXG mice and intravenously infused PBS, control CAR-T, or Rlip-CAR-T cells (**Figures 3A, B**). The experimental timeline and sampling schedule followed the Methods section.

BLI performed on days 6, 15, and 26 post-infusion showed a continuous increase in signal in the PBS group, indicating rapid tumor progression, whereas the CAR-T group exhibited markedly lower photon flux. Signal suppression was even more pronounced in the Rlip-CAR-T group and remained low throughout the 26-day observation (**Figure 3C**). ROI quantification confirmed that the average radiance of the Rlip-CAR-T group was consistently lower than that of the CAR-T group (**Figure 3D**), reflecting superior tumor control.

Survival analysis demonstrated that all PBS-treated mice succumbed within a short timeframe; while CAR-T treatment prolonged survival, the Rlip-CAR-T group exhibited a significantly greater survival benefit (**Figure 3E**).

Sequential peripheral blood sampling over three consecutive days demonstrated CAR-T cell abundance in blood was higher in the Rlip-CAR-T group at all time points (Day 1: P < 0.001; Day 2: P < 0.01; Day 3: P < 0.05, **Figure 3F**), suggesting that Rlip modification enhances peripheral blood retention.

Flow cytometric analysis revealed that Rlip-CAR-T cells exhibited significantly elevated CD47 surface expression compared to control CAR-T cells (P<0.001, **Supplementary Figure S1A**). This upregulation functionally translated into markedly reduced phagocytosis by macrophages *in vitro* (**Supplementary Figures S1C, D**), likely conferring enhanced resistance to innate immune clearance and contributing to the improved persistence of Rlip-CAR-T cells in peripheral blood.

On day 3, CD45 IHC and IOD quantification of major organs showed that CAR-T accumulation in the liver, spleen, and lungs was significantly lower in the Rlip-CAR-T group than in the CAR-T group (liver P < 0.01; spleen & lung P < 0.001), whereas no differences were observed in the heart, kidneys, or brain (**Figure 3H**), indicating reduced off-target deposition. This attenuated off-target organ accumulation correlated strongly with the peripheral blood flow cytometry data; Rlip modification increased peripheral CAR-T cell abundance, thereby reducing non-specific sequestration in normal tissues while enhancing tumor-homing capacity and probability. This optimized cellular distribution profile constitutes the pivotal mechanism underlying the superior antitumor efficacy and survival benefit exhibited by the Rlip-CAR-T group. Additionally, H&E staining revealed no obvious pathological damage in any organ (**Figure 3I**), suggesting acceptable acute histological tolerance of this modification strategy.

Together, the enhanced peripheral persistence coupled with diminished non-specific retention in normal tissues elucidates the mechanistic basis for the superior antitumor efficacy and survival advantage conferred by Rlip-CAR-T therapy.

Collectively, Rlip-CAR-T cells exhibit higher peripheral blood abundance, superior tumor control, and improved survival in the Nalm6 model while reducing off-target accumulation in the liver, spleen, and lungs. These favorable distribution profiles are likely associated with increased blood retention, thereby enhancing the therapeutic potential of CAR-T cells in hematologic malignancies.

### 3.4 Rlip nanoparticles do not alter CAR expression, activation, exhaustion, or cytotoxic function in MSLN CAR-T cells

**Figure 4:**
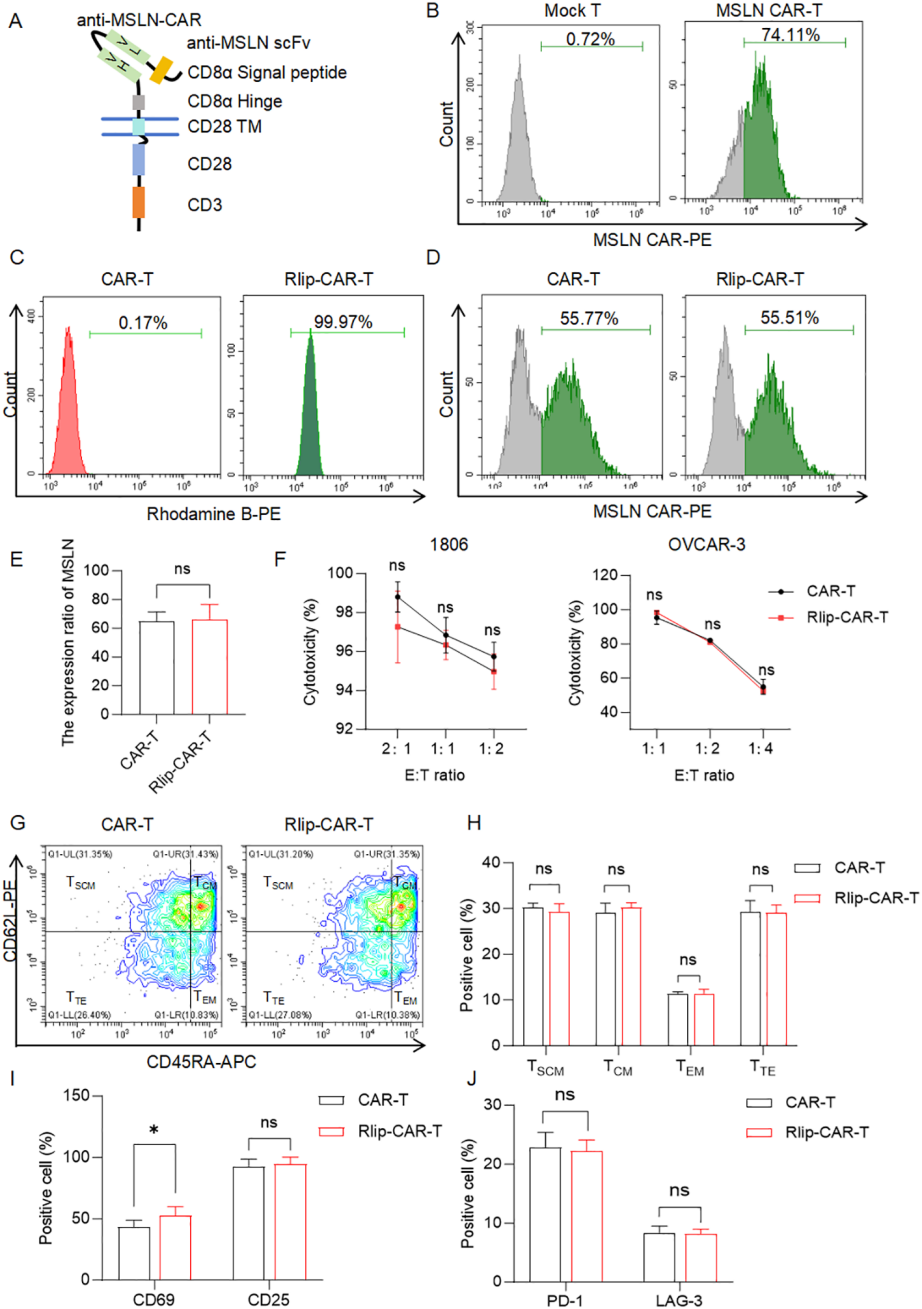
Impact of Rlip modification on MSLN CAR-T cell binding efficiency, CAR expression, and functional phenotype. **(A)** Schematic of the anti-MSLN CAR construct. **(B)** Flow-cytometry histograms showing MSLN CAR expression in untransduced human T cells versus anti-MSLN CAR-T cells. **(C)** Flowcytometry histograms of RhB positivity in CAR-T and Rlip-CAR-T cells (reflecting Rlip binding efficiency). **(D)** Flow-cytometry dot plots showing MSLN CAR expression levels in CAR-T and Rlip-CAR-T cells. **(E)** Quantitative analysis of MSLN CAR expression ratios between CAR-T and Rlip-CART cells. **(F)** Cytotoxic activity of CAR-T and Rlip-CAR-T cells against 1806 and OVCAR-3 cells at the indicated E:T ratios. **(G)** Flow-cytometry dot plots showing memory-phenotype distribution of CAR-T and Rlip-CAR-T cells stained for CD62L and CD45RA. **(H)** Quantitative analysis of T-cell memory subsets (TSCM, TCM, TEM, TTE) in CAR-T and Rlip-CAR-T cells. **(I)** Quantitative analysis of activation-marker (CD69, CD25) expression in CAR-T and Rlip-CAR-T cells. **(J)** Quantitative analysis of exhaustion-marker (PD-1, LAG3) expression in CAR-T and Rlip-CAR-T cells. All data are obtained from at least three donors and presented as mean ± SD; ns, not significant; *p < 0.05. vs. CAR-T. Cytotoxic activity **(F)** was analyzed by two-way ANOVA; all other quantitative comparisons **(E, H–J)** used two-tailed unpaired t-tests.

To validate the universal applicability of Rlip nanoparticles across tumor-targeting CAR-T cells, we generated MSLN CAR-T cells (**Figure 4A**). Flow cytometry confirmed 74.11% MSLN CAR positivity (**Figure 4B**) and 99.97% RhB positivity after Rlip incubation (**Figure 4C**), consistent with the efficient binding observed in CD19 CAR-T cells.

Rlip coating did not alter MSLN CAR expression (**Figures 4D, E**), and all functional parameters remained intact—mirroring our CD19 CAR-T findings: cytotoxicity against MSLN-positive 1806 and OVCAR-3 cells was preserved across E:T ratios (**Figure 4F**); memory subset distribution, defined by CD45RA and CD62L expression, showed no significant changes (**Figures 4G, H**); and expression of the activation markers CD69 and CD25 (**Figure 4I**) and the exhaustion markers PD-1 and LAG-3 (**Figure 4J**) was comparable between groups. These results demonstrate Rlip’s antigen-independent compatibility with CAR-T cells, preserving core phenotypes in both hematologic (CD19) and solid tumor (MSLN) contexts. This establishes Rlip as a functionally neutral, versatile strategy for CAR-T cell surface engineering.

### 3.5 Rlip surface anchoring enhances early blood retention, reduces off-target sequestration, and augments tumor enrichment of CAR-T cells in solid tumors

**Figure 5:**
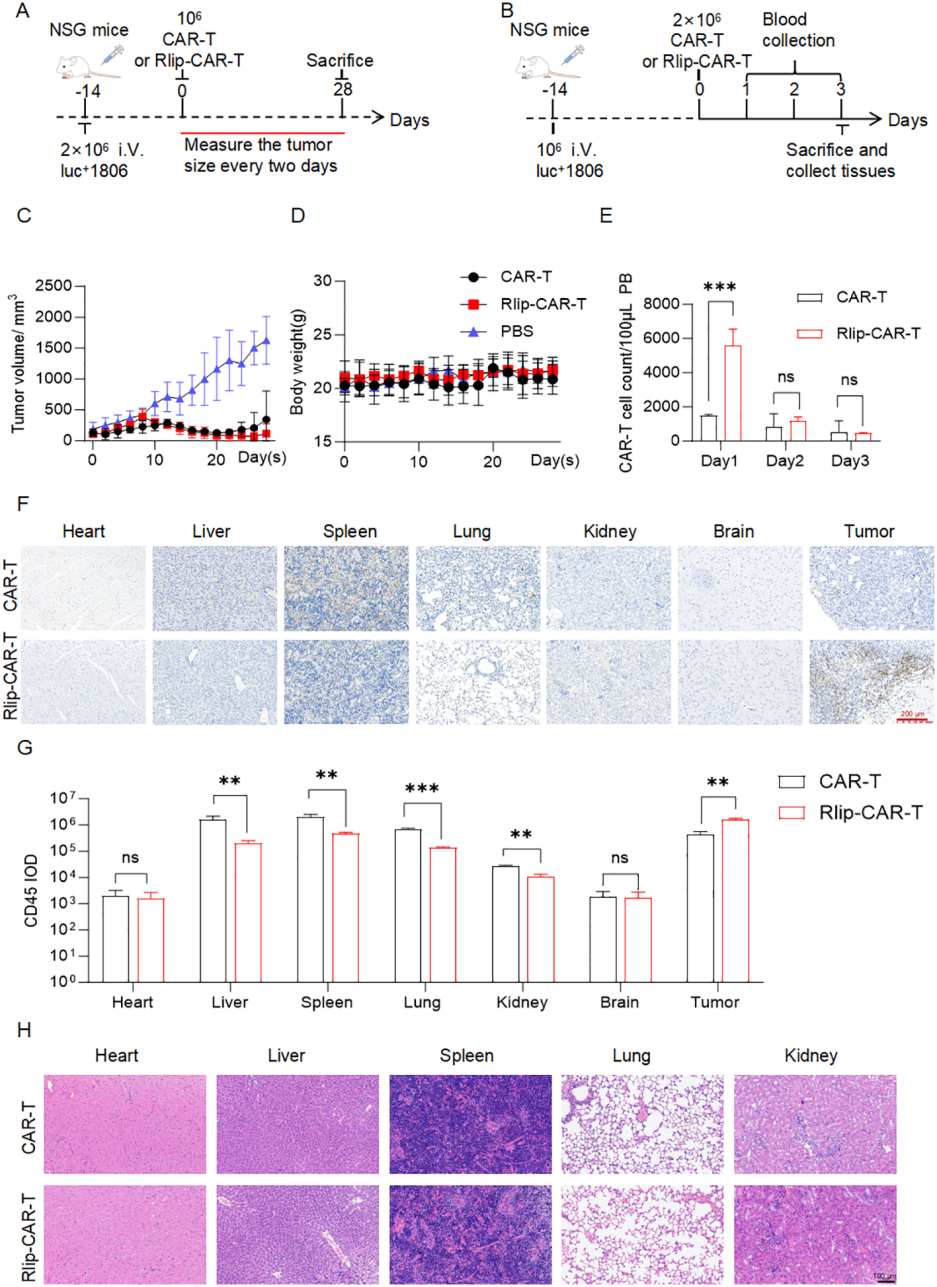
*In vivo* efficacy, peripheral blood kinetics, and tissue distribution of Rlip-CAR-T cells in a 1806 xenograft model. **(A)**. Timeline of the *in vivo* experiment: NSG mice bearing 1806 xenografts received CAR-T, Rlip-CAR-T, or PBS, with tumor size measured every two days and mice sacrificed on day 28. N = 5 mice for the PBS, CAR-T and Rlip-CAR-T groups. **(B)**. Sample-collection protocol: serial blood draws on days 1–3 post CAR-T infusion, with tissue harvest at sacrifice on day 3.N = 5 mice for CAR-T and Rlip-CAR-T groups. **(C)**. Tumor volume measurements of mice over time. **(D)**. Body weight changes of mice over time. **(E)**. Flow-cytometric enumeration of CAR-T cells in peripheral blood on days 1–3 post-infusion. **(F)**. Representative CD45 IHC images of major organs and tumor; scale bar: 200 mm. **(G)**. IOD quantification of CD45 IHC in major organs and tumor. **(H)**. Representative H&E images of major organs showing no obvious pathological damage; scale bar: 200 mm. All data are presented as mean ± SD; ns, not significant; **p < 0.01; ***p < 0.001. Statistics: two-way ANOVA for **(C)**, **(D)** and **(E)**; two-tailed unpaired t-test for **(G)**.

To evaluate the *in vivo* therapeutic efficacy, peripheral blood retention, and tissue distribution of Rlip-CAR-T cells in a solid tumor model, we established a luciferase-labeled 1806 subcutaneous xenograft model in NSG mice. Mice were intravenously infused with PBS, control CAR-T cells, or Rlip-CAR-T cells (**Figures 5A, B**). Tumor volume was measured every other day, and mice were euthanized on day 28. Peripheral blood was collected serially on days 1–3 post-infusion, and tissues were harvested on day 3, following the protocol described in the Methods. Tumor volume monitoring revealed rapid and continuous growth in the PBS group, whereas both CAR-T and RlipCAR-T groups effectively inhibited tumor progression (**Figure 5C**), demonstrating antitumor activity against 1806 xenografts. Serial blood analyses showed that CAR-T cell abundance in peripheral blood was significantly higher in the RlipCAR-T group than in the control CAR-T group on day 1, with no significant differences observed on days 2 and 3 (**Figure 5E**), indicating that Rlip modification enhances early peripheral blood retention. Consistent with observations in the systemic leukemia model, Rlip-CAR-T cells maintained elevated CD47 expression (**Supplementary Figure S1B**) and demonstrated impaired macrophage phagocytosis (**Supplementary Figures S1E, F**), supporting a mechanism of enhanced innate immune evasion that facilitates early circulatory persistence. Body weight changes did not differ significantly among the three groups throughout the study (**Figure 5F**), suggesting no acute systemic toxicity of RlipCAR-T cells. On day 3 post-infusion, CD45 IHC and IOD quantification of major organs and tumor tissue showed that the Rlip-CAR-T group had significantly lower off-target accumulation in the liver, spleen, and lungs compared with the control CAR-T group (liver and spleen: P < 0.01; lungs: P < 0.001), whereas no significant differences were detected in the heart, kidneys, or brain (**Figures 5F, G**). Notably, CAR-T cell enrichment within the tumor tissue was significantly higher in the Rlip-CAR-T group than in the control group (P < 0.01, **Figures 5F, G**). Combined with the enhanced early peripheral blood retention, these findings suggest that Rlip modification enhances CAR-T cell trafficking to the tumor site by reducing off-target organ sequestration. H&E staining of major organs revealed no obvious pathological damage in any group (**Figure 5H**), consistent with the absence of acute tissue toxicity. Collectively, in the 1806 subcutaneous xenograft model, Rlip-CAR-T cells exhibited enhanced early peripheral blood retention, reduced off-target accumulation in the liver, spleen, and lungs, and increased intratumoral enrichment, concomitant with preserved antitumor efficacy. These optimized biodistribution profiles enhance the therapeutic utility of CAR-T cells against solid tumors.

## 4 Discussion

Chimeric antigen receptor T-cell (CAR-T) therapy has revolutionized the therapeutic landscape of hematological malignancies. However, inherent limitations, including off-target organ retention, insufficient tumor infiltration, and systemic toxicity, have severely impeded its clinical translation across both hematological and solid tumors (33–37). Strategies involving the conjugation of liposomes to the surface of CAR-T cells have been extensively explored. Nevertheless, the core design rationale of existing studies primarily focuses on using CAR-T cells as targeted delivery vehicles, where liposomes encapsulate therapeutic agents to enable localized drug delivery in the tumor microenvironment (TME). By leveraging the tumor-homing capability of CAR-T cells to precisely transport drugs to lesion sites, these approaches aim to enhance antitumor efficacy through the synergistic effects of drugs and CAR-T cells (38, 39). These methods have not only refined drug distribution *in vivo* but also provided critical proof-of-concept validation for the feasibility of liposome-based carrier modification of CAR-T cells. Notably, this validated feasibility offers robust technical support for the present study. We now report an Rlip that directly tackles off-target sequestration and poor intratumoral infiltration by re-engineering the nanoparticle surface with an erythrocyte membrane. By optimizing the intrinsic *in vivo* distribution characteristics and immune evasion capacity of CAR-T cells themselves, rather than relying on drug loading for efficacy enhancement, this strategy provides a novel approach to overcome these aforementioned limitations, with its feasibility and therapeutic potential systematically validated in both hematological and solid tumor models.

As the core wrapping material on the CAR-T cell surface, the physicochemical properties of Rlip nanoparticles determine the modification efficacy and thus their *in vivo* applicability. A formulation Rlip-4 has been successfully screened and prepared in this study that exhibits a uniform spherical vesicle structure, with a hydrodynamic diameter of approximately 200 nm, a surface zeta potential of −31.0 ± 3.2 mV, and excellent colloidal stability maintained for 48 hours. These properties lay a structural foundation for resolving the core challenges of CAR-T cells: the uniform 200 nm particle size prevents recognition and clearance by the mononuclear phagocyte system (MPS) caused by particle aggregation. Meanwhile, the strongly negative zeta potential (absolute value ≥ 30 mV) (40) not only maintains suspension stability and prevents aggregation through interparticle electrostatic repulsion, but also reduces non-specific adsorption between CAR-T cells and vascular endothelial cells, thereby fundamentally mitigating the risk of off-target organ retention. The robust colloidal stability of Rlip-4 over 48 hours, coupled with stable retention on the CAR-T cell surface under cell culture conditions, ensures that the modification layer persists during systemic circulation, sustaining the optimization effects. Collectively, these physicochemical and interfacial properties of Rlip-4 are precisely tailored to meet the requirements of CAR-T cell surface modification, laying a critical foundation for subsequent optimization of *in vivo* distribution and therapeutic efficacy.

When evaluating the feasibility and efficacy of the Rlip modification strategy, besides verifying the physicochemical properties of nanoparticles and the optimization effects of *in vivo* distribution, the integrity of the functional phenotypes of CAR-T cells themselves is also a core consideration. If the modification process impairs the memory potential, activation status, or viability of cells, improved distribution characteristics alone cannot achieve desirable antitumor outcomes. Therefore, we systematically evaluated key phenotypic markers, namely CD62L, CD45RA, PD-1, LAG-3, CD69, and CD25, to assess CAR-T cell function across three critical dimensions: subset composition, activation state, and exhaustion level.

CD62L and CD45RA define T-cell subsets that dictate *in vivo* persistence and proliferative capacity. The TSCM and TCM subsets are critical for durable therapeutic efficacy, whereas CD62L^+^CD45RA^+^ naive, TEM, and TTE cells confer only transient effects (41–45), PD-1 and LAG-3 are canonical exhaustion markers whose upregulation correlates with functional impairment and diminished antitumor activity (46–49). CD69 and CD25 reflect activation status: CD69 as an early activation indicator and CD25 as the IL-2 receptor α chain essential for proliferation and effector function (41, 50, 51).

Flow cytometry and cytotoxicity assays confirmed that Rlip modification preserved core CAR-T cell functional phenotypes. CAR expression remained stable post-modification, and the proportions of TSCM and TCM subsets were unchanged, ensuring sustained proliferative potential. Normal CD69 and CD25 expression indicated unimpeded activation, while stable PD-1 and LAG-3 levels demonstrated the absence of premature exhaustion. Critically, Rlip-modified CAR-T cells exhibited cytotoxic activity against CD19-expressing (Nalm-6, Raji) and MSLN-expressing (1806, OVCAR-3) target cells comparable to unmodified controls, substantiating the functional safety of this modification strategy at the cellular level.

*In vivo* studies validated the capacity of Rlip modification to optimize CAR-T cell biodistribution and antitumor efficacy. In the Nalm-6 leukemia model, Rlip-CAR-T cells demonstrated enhanced peripheral blood persistence, leading to significant reductions in tumor burden and prolonged survival. CD45 immunohistochemistry confirmed decreased off-target accumulation in the liver, spleen, and lung, with no histopathological abnormalities observed in major organs, corroborating the absence of overt tissue damage associated with the modified cells. Similarly, in the 1806-luc solid tumor model, Rlip modification increased early peripheral blood retention (Day 1, P < 0.001) and promoted tumor infiltration (P < 0.01) while maintaining minimal non-specific organ sequestration. Mice in this model maintained stable body weight, and histopathological analysis revealed no treatment-related abnormalities. Across both tumor models, these findings demonstrate that Rlip-modified CAR-T cells preserve potent antitumor activity while exhibiting an improved biodistribution profile characterized by reduced off-target organ accumulation and preserved tissue integrity.

The advantages demonstrated by these *in vivo* results are highly consistent with the established findings in the nanocarrier field. Previous studies have clearly shown that liposomal carriers, by virtue of their unique vesicle structure, can more efficiently deliver payloads to tumor tissues while reducing accumulation in normal organs such as the liver and intestines (52, 53). In this study, the liposomal scaffold of Rlip may similarly contribute to the reduced off-target organ distribution observed for Rlip-CAR-T cells. Furthermore, multiple studies have confirmed that nanocarriers coated with red blood cell membranes can mimic the immune evasion characteristics of natural red blood cells, exhibiting significantly prolonged *in vivo* circulation time compared with conventional liposomes and showing reduced distribution in immune organs, including the lungs and spleen (22, 23). This immune evasion is partly mediated by membrane proteins such as CD47, which transmits a “Don’t eat me” signal through interaction with SIRPα on macrophages, thereby inhibiting phagocytic clearance. Flow cytometric analysis revealed significantly higher CD47 surface expression on Rlip-CAR-T cells compared to control CAR-T cells, providing a mechanistic basis for the enhanced peripheral persistence observed in our study. The prolonged peripheral blood retention of Rlip-CAR-T cells aligns with these reported properties of red blood cell membrane-coated systems. Together, these observations suggest that the liposomal scaffold and red blood cell membrane components of Rlip may collectively contribute to the improved biodistribution profile of CAR-T cells, potentially by reducing non-specific organ accumulation and extending circulatory persistence, ultimately supporting the dual goals of mitigating off-target sequestration and enhancing tumor accumulation.

## 5 Conclusions

In summary, this study demonstrates that surface modification of CAR-T cells with an erythrocyte membrane–liposome effectively reduce their off-target sequestration in normal organs and significantly enhance the enrichment of CAR-T cells at tumor sites. We identified Rlip-mediated membrane engineering as a key strategy to address the intrinsic distribution bottlenecks of CAR-T therapy. This technology optimizes the *in vivo* distribution profiles of CAR-T cells by mitigating non-specific organ retention, thereby providing a theoretical basis for overcoming the distribution-related obstacles of CAR-T therapy and improving its therapeutic efficacy in both hematological malignancies and solid tumors.

## Data Availability

The original contributions presented in the study are included in the article/**Supplementary Material**. Further inquiries can be directed to the corresponding authors.
