# Supplementary material for: Erythrocyte membrane–liposome coating sustains circulation stability and targeted tumor therapy of CAR-T cells
Source: Front Immunol. 2026 Apr 2;17:1799107. doi: 10.3389/fimmu.2026.1799107 (PMC13083064; doi:10.3389/fimmu.2026.1799107)
Supplement: Supplementary file 1 [file Table1.docx]

Supplementary

Erythrocyte Membrane–Liposome Coating Sustains Circulation Stability and Targeted Tumor Therapy of CAR-T Cells


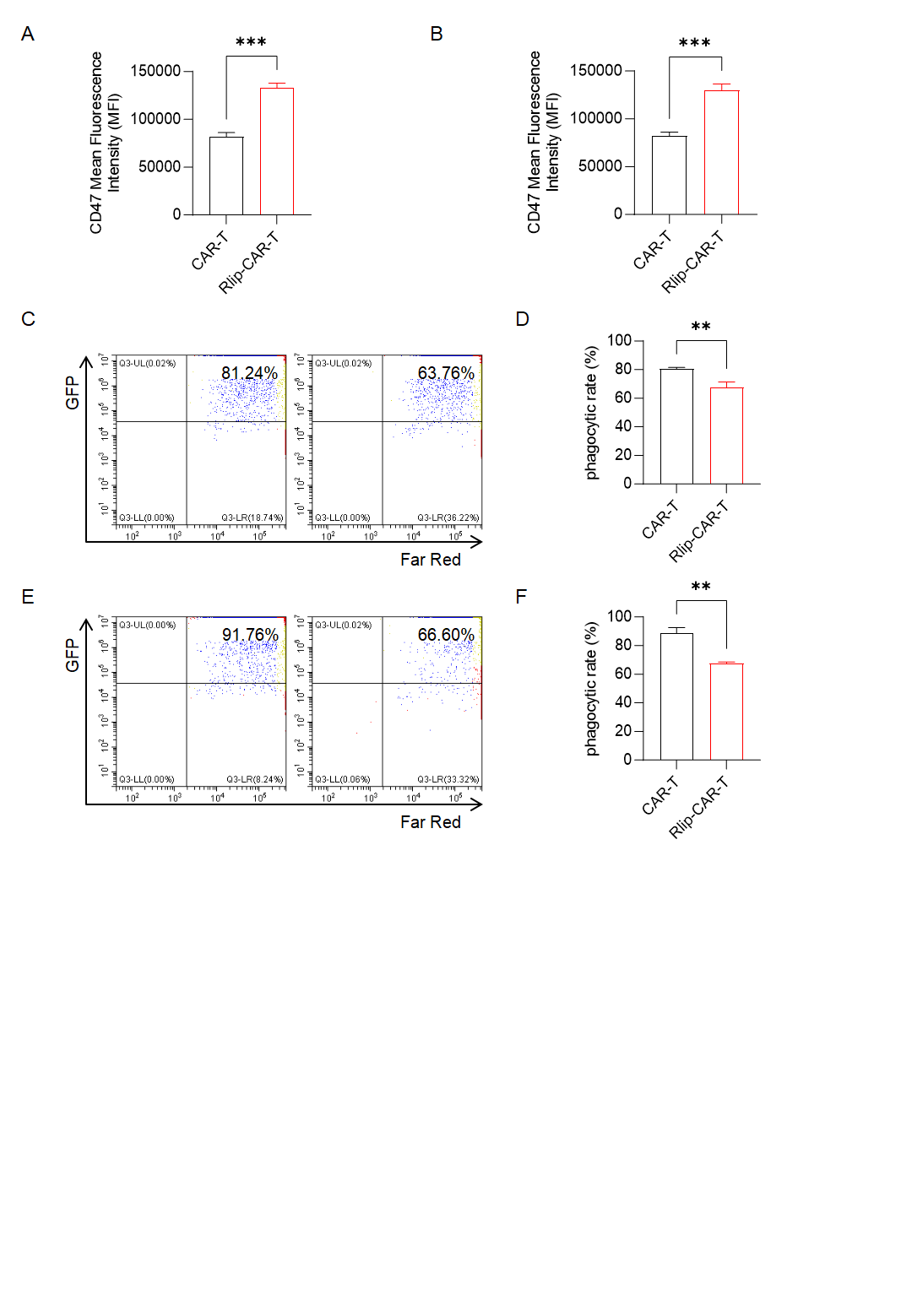


Fig. S1.Figure S1. CD47 surface expression on Rlip-modified CD19- and MSLN-targeted CAR-T cells and their phagocytosis by macrophages.

(A) CD47 surface mean fluorescence intensity (MFI) on CD19-CAR-T and Rlip-CD19-CAR-T cells.(B) CD47 surface MFI on MSLN-CAR-T and Rlip-MSLN-CAR-T cells.(C) Representative flow-cytometry dot plots showing phagocytosis of CD19-CAR-T and Rlip-CD19-CAR-T cells by macrophages.(D) Phagocytosis rate of macrophages on CD19-CAR-T and Rlip-CD19-CAR-T cells.(E) Representative flow-cytometry dot plots showing phagocytosis of MSLN-CAR-T and Rlip-MSLN-CAR-T cells by macrophages.(F) Phagocytosis rate of macrophages on MSLN-CAR-T and Rlip-MSLN-CAR-T cells.All data are obtained from at least three donors and presented as mean ± SD; **p < 0.01; ***p < 0.001. All quantitative comparisons were analyzed by two-tailed unpaired t-tests.
